# Supplementary material for: The Neuropathy Tracker—A mobile health application for ambulatory and self-administred assessment of neuropathy
Source: PLOS Digit Health. 2025 Feb 12;4(2):e0000725. doi: 10.1371/journal.pdig.0000725 (PMC11819570; doi:10.1371/journal.pdig.0000725)
Supplement: Attachment — Submitted filename: Answer.Letter.to.Reviewers.pdf [file pdig.0000725.s001.pdf]

Review of PDIG-D-24-00371: The Neuropathy Tracker – A mobile health application for ambulatory and self-administered assessment of neuropathy

Answer to Reviewers

Overall, we would like to thank the reviewers for their very detailed and constructive feedback, which in our opinion has helped improve the paper significantly. Below you will find a point-to-point answer to the different points raised by each reviewer. Moreover, in the marked-up version of the document you will see that the paper has been updated and improved throughout.

In this document, the black text is the original comment by the reviewers, and this indented text in italic red is our answer to the comment.

Reviewer #1

The manuscript is well-written, clear, and presents an interesting approach to addressing an important issue in neuropathy detection and monitoring. The use and detailed description of methodological approach of the user centered design is a strong characteristic of the paper, as well as the depiction and detailed description of how the app works.

Thank you for this feedback, which reveals that the main purpose of the paper – namely describing the user-centered design of the app – seems to be clear from the manuscript.

However the use of this methodology does not "ensure a high degree of clinical validity and usability" as stated in the manuscript. So does not the feasibility study conducted, given the small number of subjects (N=17) and the selection bias (once all participants were recruited at the same clinic). In order to sustain such a claim a much larger and rigorous validation study would be necessary.

We completely agree with this and a larger and much more rigorous validation study is ongoing. However, we find it relevant to include this small feasibility study in this paper to provide some initial and preliminary insight into the workings of the proposed solution in the clinic.

A more cautious and tentative language would be advised to describe the results of the study, which is relevant though preliminary.

We agree, and the presentation of the study and results have been rephrased in several places, all highlighted in the marked-up version of the revised manuscript.

A major problem of the study is the fact that the total neuropathy score clinical, which is a central result, was assessed by only one person who is described to be a newly trained non-clinical research assistant. This limitation compromises the rigor of the validation study.

We agree, and this problem has been highlighted as part of the study's limitations.

Reviewer #2

Overall

The authors describe the creation of the "Neuropathy Tracker", a mobile health tool designed for ambulatory neuropathy self-assessment. This represents overall, a reasonable initial feasibility study, although clearly additional study would be needed before releasing this broadly to patients.

We agree and a larger and much more rigorous validation study is ongoing. We have included a sentence on this in the conclusion of the updated manuscript.

I commend the authors on the clear degree of detail, as often the papers for mobile health applications do not give a clear sense of their actual contents and function

Thank you. This was exactly the purpose of this paper, to describe in more detail the user-centered design methods and technology underpinning the app. We agree that this is unfortunately often missing which is important for the more technically oriented audience of PLOS.

Overall, however, my greatest concern is that the paper has somewhat excessive information in parts of the methods section, as well as a lack of sufficient detail in the area of the results. Some of the intense technical detail would benefit from being moved to a supplement, particularly regarding the computational software strategy (although the detail around the decision-making behind the project is excellent).

Based on the reviews, we have added substantially more details and discussion on the area of the results from the feasibility study. We have also carefully revised the technical part of the manuscript, but since the main purpose and contribution of this paper is to describe the UX design and technical implementation of the Neuropathy Tracker technology, we have kept the core technical details for the more technically-oriented reader of PLOS.

The results section come on the other hand, has only a general correlation metric while I would like to see a little bit more of the subtest by subtest metrics.

I have some other concerns specific to methodology:
With respect to methodology, please consider explaining why TNSc was chosen as the comparison standard rather than UENS, given that the Neuropathy Tracker is more closely based on UENS.

This was also the subject of considerable discussion. However, we decided to use TNSc since it is a more valid "external" comparison already used in the clinic. Moreover, the app cannot use the TNSc method, since this is not designed to be used for patient self-assessment. This has now been explained in the manuscript.

In this initial technically-focused paper on the Neuropathy Tracker App, we focus on showing how the summary score could reflect the neuropathy burden. Therefore, we decided to use an external validation approach showing a correlation with an established clinical neuropathy score.

For the further validation of the tool, we will go into a detailed validation of subscores using internal validation towards clinical UENS by professionals and more comprehensive external validation. If we presented an internal validation of the app against UENS, we would be severely underpowered in this study to document correlations in most subscores.

Discuss potential biases in the study design, such as the order effect of always performing the Neuropathy Tracker assessment before the TNSc.

The order of the two tests in the study was quite deliberate. This has now been explained as part of the "Methods" section.

Specific comments

• Abstract:
  ○ The abstract is clear in its overall structure and content.
  ○ Include more specific results from the feasibility study, such as the correlation coefficient and p-value.

As described below, the T-test has been replaced by a Bland-Altman plot and a Concordance Correlation Coefficient (CCC) test, which is now also reported in the abstract.

• Author Summary:
  ○ Author summary is accurate and high-quality

• Introduction:

○ I enjoy the degree of detail in the introduction as a neurologist, however I wonder if there may be some excessive detail given about the many different neuropathy scores and questionnaires that distracts from the flow.

We have reduced this part a bit, but still kept an introduction to the scores used later in the paper.

○ Additional discussion of the mhealth landscape in which this project is situated may be more appropriate (which I know is included in the discussion, but could be alluded to earlier)

An introduction to the mHealth landscape (a very few set of paper) is now shortly added to the introduction while the more detailed discussion on related apps is still kept in the discussion section, where more space can be allocated to this.

• Methods:

○ The design process is clearly explained, and reasonable.

○ The figures are useful and give a clear sense of the structure and function of this application, however the authors could condense the description of the UX design process, focusing on key decisions and rationales rather than detailing every screen.

We have condensed this section a bit. However, other reviewers highlight the detailed description of the app design as a contribution to the paper – something we agree to and in our opinion is the major contribution. Therefore, we have kept the description as part of the paper.

○ "To avoid mixing self-reported symptoms and more objective findings, this question has been placed first in the examination."

§ This is very clever and an excellent idea.

○ The use of the vibration function of a smart phone is also similarly brilliant, although with caveats as the authors note.

§ I would add discussion of issues with variable vibration INTENSITIES in addition to the discussion of variable frequencies.

None of the patients changed the default settings of the vibration, even though this is an option in the app. This has now been explained in the description of the study and observations on the use of the vibration actuator are added both to the "Results" and "Limitation" sections.

○ The level of technical detail provided is excellent, however, I wonder if some of this detail is better suited for the supplemental portion.

As mentioned above, an important contribution of the paper is the technical details, which are targeted at a more technical / engineering audience of PLOS. Hence, we

have kept this in the main part of the paper. We will, however, talk to PLOS about adding some supplementary material to the final version of the paper, if more details are needed.

• Results:

○ Expand this section significantly to provide more detailed analysis:

§ Include a breakdown of scores for each subsection of the assessment (e.g., pin-prick, vibration, motor tests) comparing the Neuropathy Tracker results to the TNSc.

§ Provide statistical analysis for each subsection, not just the overall score.

§ Include a table showing mean scores, standard deviations, and p-values for each subsection.

Doing an analysis per subsection is a very good idea. We have investigated this suggestion but have found it to be less feasible with the current design of the study. There is no clear mapping from the assessment done in the Neuropathy Tracker to subsections in the TNSc scale.

Moreover, when designing the Neuropathy Tracker App we decided to make a two-step validation. The present paper focuses on the app design, and how we instruct the patient to perform a self-evaluation of symptoms and signs. We included the limited external validation to show the readers how we aim to use the summary neuropathy score from the app. We did not power this limited validation for anything beyond this. As a result, we only report the external validation using TNSc and do not claim the app subscore from the patient self-evaluation to correlate with the examination performed in the clinic. We do not find it proper to perform subscore comparisons in this paper, as the two neuropathy grading tools are not comparable in examination technique.

In an upcoming paper, we focus on documenting the validity of patient-reported outcomes by conducting a full internal and external validation against TNSc, UENS, and QST in a larger cohort.

§ Specifically address the validity of using the phone's vibration function compared to a tuning fork.

We did not make this comparison during the test, so this cannot be reported. But, as mentioned above, we have added more details on the use of the vibration actuator on the phone.

○ Clarify the statistical methods used, explaining what exactly is being compared in the t-test and why this test was chosen.

Based on the reviewers' very helpful comments, the statistics have now been redone and now include both a Pearson Correlation Coefficient, a Concordance Correlation Coefficient (CCC), and a Bland-Altman plot. All of this is explained in details.

○ Consider adding a Bland-Altman plot --- or other detailed metric of the authors' choosing --- to visualize agreement between the two assessment methods.

This is excellent input and we have adopted this. Figure 11 (the old scatter plot) has now been replaced by a Bland-Atlman plot and is presented and discussed in the manuscript, as highlighted in blue. Furthermore, a Concordance Correlation Coefficient (CCC) value is calculated and discussed.

• Discussion / Limitations:

○ It is alluded to, but the authors should clearly highlight the limitation that the small sample size likely limits some of the subsection by subsection analysis that would be necessary to fully validate this test.

The "Limitations" section has now been expanded to discuss the limitations of the feasibility study further.

○ In addition, the limitations section should highlight the lack of test-retest validation for this tool.

The "Limitations" section has now been expanded to also mention the lack of test-retest in the present study.

○ Address accessibility features for users with visual or motor impairments.

The "Limitations" section has now been expanded to also mention the lack of testing for accessibility in the present study.

○ Discuss what score threshold might be clinically meaningful for neuropathy detection or monitoring.

This study is too small to use the scores and hence identify thresholds for clinical conditions thereby suggesting a diagnostic property of the tool. This point has been added to the Discussion section of the paper now.

We intentionally avoid framing the tool as a diagnostic tool. Instead, we have tried to consistently frame it as a reporting tool for symptoms and findings.

We have, however, discussed this important question in detail and when planning for the clinical validation study to come we will include this. We find it important to include it in the discussion of that upcoming paper.

○ Address how the tool's performance might vary across different severities of neuropathy.

The Bland-Altman plot revealed a small proportional bias since the app tends to under-score on low scores and over-score on high scores. A discussion of this has now been added to the paper. Analysis of bias will also be part of our larger clinical validation study.

Reviewer #3

The manuscript presents a promising and innovative approach to assessing neuropathy through the development and validation of the Neuropathy Tracker, an app designed to enable self-assessment by patients outside of clinical settings. One of the key strengths of this study is its focus on user-centered design, ensuring that both patients and clinicians are involved in shaping the usability and functionality of the app. The integration of digital health technologies, such as the Neuropathy Tracker, represents a significant step forward in improving patient autonomy and early detection of neuropathic symptoms, which can lead to better management and outcomes. The authors should be commended for addressing a critical need for accessible, scalable tools in neuropathy care and for producing strong initial results with a high correlation between the app's scores and those obtained from the well-established TNSc assessment. The authors embrace of a platform neutral technology (Flutter) demonstrates their vision for scalability and universal use. This work has the potential to make a meaningful impact on the way neuropathy is monitored and managed, particularly in the comfort of patients' domicile, and paves the way for future studies to further validate and refine the tool.

Thank you for this overall assessment of the contribution of the manuscript. This was exactly the purpose of this paper, to describe in more detail the user-centered design methods and technology underpinning the app and this novel approach to ambulatory assessment of neuropathy. We are also glad to see that the small feasibility study is highlighted as a positive contribution, despite its apparent limitations. We find that this feasibility study adds to the paper and we have decided to keep it while revising its description based on all the very useful comments provided below.

Primary observations

1. Scope of approval ("Quality Study") and Inconsistency with terminology used for study.
Issue: The manuscript refers to the study as both a Validation Study and a Feasibility Study in multiple locations, while the Ethics Committee approval is explicitly for a Quality Study. It appears like these terms are being used interchangeably. A Quality Study typically focuses on usability, functionality, and performance, not clinical validation. However, the manuscript

includes aspects of concurrent validity and comparisons with TNSc scores, which extend beyond the typical scope of a Quality Study and venture into clinical validation which is further affirmed in the conclusion. This discrepancy raises concerns about whether the study remains within the bounds of the ethical approval granted.

Suggestion:
1. Clarify the Study's Scope: Ensure that the manuscript consistently uses terminology aligned with the Ethics Committee approval for a Quality Study. If it was approved as a Quality Study, focus should be on usability, functionality, and user satisfaction, not on validation against clinical tools. A feasibility study may be construed as a type of quality study. Line 74 clearly states the objective of the study (the introduction of the term "evaluation study" in this line further adds to the confusion. The word "evaluation" may be dropped here. Based on this objective, it is recommended that the study be referred consistently as "Feasibility study" and add one sentence in appropriate location to justify why a feasibility study may be considered a Quality study.

> *This is indeed very valuable input and a very useful observation. We have throughout the paper now consistently described the study as a "feasibility" study and how this is within the scope of the ethics approval. More information on the wording from the ethics committee is also included, to explain what is meant by a "quality" study in a Danish context. The changes are highlighted in blue in the revised manuscript.*

2. Reframe the Study: Emphasize usability and performance-related aspects and avoid making strong claims about clinical validation or concurrent validity. Consider downplaying clinical comparisons with TNSc scores. If this approach is taken then it should be reflected in Abstract, methods, discussion and conclusion.

> *We have updated the entire manuscript to reflect these changes, incl. in the abstract, methods, discussion, and conclusion. Changes are highlighted in blue. Note that the statistics have been changed based on input from Reviewer #2.*

3. Seek Further Approval if Necessary: If clinical validation is a key goal of the study, the authors should seek additional ethical approval for a Validation Study or a Clinical Study to align with the scope of the analysis and avoid ethical discrepancies.

> *We are in the process of running larger-scale clinical validation studies, which have a separate set of ethics approvals.*

NOTE: The tool essentially introduces a new blended score that is being demonstrated as linearly correlating with TNSc. If the primary objective is to validate this new score provided by the App (as stated) then the scope will definite creep into Clinical validation and will require substantial evidence work.

We are currently conducting a larger clinical validation study which has its own ethics approval. This other study is, however, outside the scope of this manuscript.

2. Mention of analyzing predictive power in Validation methods section and not discussing it anywhere else.
Issue: Line 114 states “The predictive power of the new method is analyzed using correlation and linear regression”. There is no further mention of Predictive power anywhere else in the manuscript.
Suggestion: If the intent is to predict TNSc based on App score then then it will have to be explicitly mentioned in the Validation methods section and subsequently presented as a model (regression equation? along with measures for statistical robustness in the results section. It should also be discussed in the discussion section. If this is not the intent, it is recommended that the sentence be dropped from the Validation methods section. This could also be presented as a future study avenue due to the robust correlation.

The goal of the feasibility study was not to make regression models for predictions. It was merely to establish early evidence on “functionality”, i.e., as to what degree the scores obtained by the app align with the TNSc scores. However, based on input from R#2 a better way to establish this “concurrent validity” is to use a Bland-Altman plot and a CCC score, which to a much better degree illustrates and reveals the degree of agreement between the two scores.

3. The objective of paired t-test is unclear
Issue: The Concurrent validity section has the following sentence in lines starting at line 386. “The plot shows a clear linear correlation with a Pearson correlation of ρ = 0.861 and a paired t-test showed a statistically significant p-value of p = 0.01”. What is the objective of paired t-test in this context? A paired t-test is used to compare the mean of two sets of data. In this case, the sentence as written (“a paired t-test showed a statistically significant p-value of p = 0.01”) implies the mean of scores are statistically different. This is also inferable from the plot.
Suggestion: Drop the mention of paired t-test as it appears to be irrelevant for this study. If there is a reason for its existence, then please discuss the significance of the result in results and discussion sections. Also, if you plan to retain, correct the mention of “p = 0.01 to p < 0.01”.

We fully agree. This whole section has been replaced with the discussion of the Bland-Altman plot and CCC score.

4. Number of steps involved in the user interface
Issue: Line 248 mentions that the test involves 39 steps. If the objective of the study is Quality Study (as approved by the Ethics Committee) then elaborating on the user experience around the steps would be a valuable outcome of your efforts.
Suggestions: I would encourage including any additional metrics that might have been observed around the actual user test. For example, average time, steps, completed / not completed, etc., and any qualitative information. Any evidence as to why the number of steps involved is a

barrier for usage will be beneficial. As such, the Usability section makes a claim about usability issues without any measures as evidence.

> *This is valid input and the paper has now been updated with much more detailed information on the usability part. However, the usability evaluation was based on qualitative methods using participant observation. We did not time the test (which is now also explained) since the patients were told to spend as much time as needed on the test. So - timing would work against this instruction. This is, however, a very good input for the design of our current validation study.*

5. Conclusion (including the abstract) is very strong on the clinical strong on the clinical aspects
Issue: The conclusion states “These results allow for using the Neuropathy Tracker in clinical diagnosis and treatment of neuropathy as part of clinical treatment in a clinic”. This claim is extremely strong and inconsistent with the approved intent of the study (Quality Study). Statement about utility for diagnosis and treatment require clinical validation.
Suggestions: The authors have done commendable work in demonstrating that an off the shelf mobile device can be used to self-assess probable Neuropathy and seek further professional help. It is suggested that this study be emphasized as evidence of this aspect and not make any claim about diagnosis and treatment – which are extremely strong and not substantiated in this manuscript. The potential for being used in diagnosis may be discussed in future studies section. If this app is to be used for diagnosis, then it will have to be reviewed as a Sofware as a Medical device and the process is well beyond the scope of this work. The feasibility aspect of the study should focus on user’s ability to self assess. If so, then this study may be presented as a Quality study. If authors agree, the please deemphasize diagnosis and treatment aspects in conclusion. Please make sure the abstract is also edited accordingly.

> *We fully agree and this sentence (plus some others related to it) has now been rephrased in the entire manuscript.*

Minor observations

1. Correct Typographical Errors
The title and at least one other reference has the term "self-administred" instead of "self-administered." Ensure a thorough proofreading is done before submission. There are a few places where the grammar could be improved. Please review with a grammar correction tool if available.

> *Thanks for pointing this out. We have consistently used a grammar correction tool in the updated version of the manuscript, and thorough proofreading of the final manuscript will take place.*

2. Tables and figures placement
Issue: Some tables and figures appear far from where they are first referenced in the text, which can interrupt the flow of reading. For example, Fig 1 is embedded in the Introduction section,

whereas, it is referred within a sub-section (User-centered design process) that is within the next section (Methods and Material)
Suggestion: Recommend moving tables and figures closer to the point where they are first mentioned to improve readability. Ideally these should be place right above or below the paragraph with the first reference.

> *LaTex has its own mind on where to put figures and tables, but we have tried to rearrange them in the updated manuscript. Please see the version of the manuscript without markup, since the markup'ing moves the figures around.*

3. Potential confusion because of the terms “Phase I and Phase II”
Issue: While it is clear that the authors are referring two stages of validation, given the specific use of terms Phase I and Phase II in regulatory process, this may lead to unnecessary confusion.
Suggestion: Recommend using terms like “Part 1, Part 2”, or “Stage 1, Stage 2” instead of the term “Phase”

> *This is a very valid point and we have adopted the use of “part” instead of “phase”.*

4. Clarity on role of research assistant
Issue: It is not clear if same research assistant conducted TNSc evaluation and monitored while patients conducted self-evaluation. If that is the case, the situation may lead to Observer bias.
Suggestion: If multiple research assistants with specific roles were involved then mention it explicitly. If not, call out the potential for observer bias in the limitations section.

> *This is also a very valid point (and yes, it was the same person). This has now been clarified and discussed in the “Limitations” section.*

5. Usage of the term “Digital Phenotyping Tool”
Issue: The term "digital phenotyping" may not be entirely appropriate, as digital phenotyping typically refers to the continuous, passive collection of digital data (like behavior, movement, or communication patterns) to infer health-related outcomes, especially in mental health or chronic diseases. In contrast, the Neuropathy Tracker seems to focus on ambulatory self-assessment, which suggests it is more likely collecting specific, deliberate health-related measurements rather than passively tracking behaviors or traits.
Suggestion: Considering using more appropriate terms like “Digital Health Assessment Tool”, “Digital Self-Monitoring Tool”, “Digital Biomarker Collection Tool”, etc.

> *This is a very valid observation. The term “Digital Phenotyping” makes little sense in this work and has been removed from the manuscript. We now refer to the work as a mHealth app for ambulatory self-assessment.*

6. Lack of clarity on material required for study

Issue: The section “Validation methods” refers to “required materials” for the study but does not provide a listing.
Suggestion: To enable easy repeatability, provide a listing or required materials.

This was actually just the safety pin, which now has been explained.

7. Mention test and test result while referring p value
Issue: The line 411 mentions “The study showed a statistically significant ($p < 0.05$) degree of concurrent validity between the overall …”. As such, the p value limit is not available in the Validation methods section.
Suggestion: Include the significance level in line 387 in association with pearson correlation coefficient. The name of the and the result should be mentioned along with the significance value should be mentioned here. Recommend rephrasing along the following lines: “The study showed a statistically significant degree of concurrent validity (Pearson correlation of $\rho = 0.861$, $p < 0.05$) between the overall …”

Thanks for the input. We have followed this when reporting on the statistics throughout the paper.
